# Preschool teachers’ STEM pedagogical content knowledge: A comparative study of teachers in Greece and Turkey

**DOI:** 10.3389/fpsyg.2022.996338

**Published:** 2022-11-08

**Authors:** Ali İbrahim Can Gözüm, Stamatios Papadakis, Michail Kalogiannakis

**Affiliations:** ^1^Dede Korkut Faculty of Education, Kafkas University, Kars, Turkey; ^2^Faculty of Education, The University of Crete, Crete, Greece

**Keywords:** STEMPCK, preschool teachers, Greece, Turkey, STEM

## Abstract

This study compares the STEM Pedagogical Content Knowledge of Greek and Turkish preschool teachers. The present research is a comparative descriptive study that aims to determine the STEM Pedagogical Content Knowledge of preschool teachers from Greece and Turkey. A descriptive survey model, a method used in quantitative research, was used as this study’s primary research method. The STEM Pedagogical Content Knowledge Scale (STEMPCK) was used in this study. Six hundred sixty-nine preschool teachers - 104 Greek and 565 Turkish teachers - participated in this study. The STEMPCK Scale’s construct validity and reliability were tested using this study’s data set, which was found to be both valid and reliable. No significant difference was found between the STEMPCK scores of Greek and Turkish preschool teachers. The significant differentiation of STEMPCK scores based on whether the teachers had received any STEM training is discussed in light of the relevant literature. This study determines and compares STEMPCK among preschool teachers from disparate countries such as Greece and Turkey and is expected to contribute to the literature.

## Introduction

The skills of the age of individuals should have been known as 21st-century skills. Science, technology, engineering, and mathematics (STEM) education stands out as an essential practice in developing 21st-century skills (Marsono et al., 2019). By combining science, engineering, technology, and mathematics disciplines, STEM education enables individuals to identify real-life problems, develop alternative and practical solutions, and offer creative, original solutions which are crucial for the 21^st^ century (Cooper and Heaverlo, 2013; Moore et al., 2014).

STEM education is included in the educational practices of many countries from early childhood through to higher education. STEM education practices are critical in early childhood (Moomaw and Davis, 2010). According to Eshach and Fried (2005), implementing STEM activities in early childhood significantly affects children’s attitudes toward science. Children who did not practice STEM activities in their early years lose interest in science later (Allen, 2016) and may not choose STEM-related fields in their academic careers (Brophy et al., 2008). The concept of providing STEM education to children in the fields of science, technology, engineering, and mathematics (Sullivan and Bers, 2016) supports children’s cognitive, psychomotor, social, and affective development (Torres-Crospe et al., 2014; Clements and Sarama, 2016) by assisting their reasoning skills and thinking (Gonzalez and Freyer, 2014; Mercan and Kandır, 2022).

Children discuss the present world’s critical problems in their early years as part of STEM education. For example, children’s scientific thinking and problem-solving skills are supported by examining health problems such as energy, environmental pollution, and epidemics (Bybee, 2010). What matters here is to choose a qualified STEM program (MacFarlane, 2016) and for the teacher to apply that program to have the pedagogic knowledge and proficiency required for STEM (Margot and Kettler, 2019). Park et al. (2017) said in their study that preschool teachers believe the STEM approach should be adopted in the early years but emphasized that teachers cannot implement STEM activities in educational settings and should be supported. Teachers should be taught how to use effective teaching methods to implement STEM activities, and their STEM knowledge and skills should be backed by pedagogic field experience (Lichtenberger and George-Jackson, 2013). Related studies (Reimers et al., 2015; McDonald, 2016; Mercan and Kandır, 2022) revealed that supporting both the content of STEM disciplines and the development of teachers’ pedagogical knowledge positively affected in-class STEM practices and supported the children’s development. Preschool teachers’ content and pedagogical knowledge for STEM disciplines must be supported (Kennedy et al., 2008).

The pedagogical knowledge of preschool teachers emerges in their qualified and effective use of teaching practices while applying STEM activities to children (Hudson et al., 2015). Content knowledge of teachers for STEM activities forms the basis of scientific understanding to be gained by children in STEM activities. However, the critical point to be considered in content knowledge and pedagogy knowledge for STEM education is teacher training. Including STEM content and pedagogical knowledge in the programs where teachers receive pre-service training is a critical element of STEM education (Ball et al., 2008). In the context of teacher education programs, content and pedagogical knowledge adequacy related to STEM are discussed (Kaya and Elster, 2019). Thus, in-service training programs are implemented to support teachers’ STEM content knowledge and pedagogical knowledge. Many countries have established policies to promote STEM education and teachers’ professional development in STEM (Johnson, 2012). Countries such as Canada, the United States, and Australia have reported an increased need for teachers trained in STEM so that STEM education can be given effectively and proficiently in classrooms (Stohlmann et al., 2014).

There is no specific standard or content agreed upon in the teacher training policies of countries for STEM education. Therefore, it is thought that countries should focus on the difficulties and problems they experience in the STEM education process to meet their expert teacher needs. Implementing STEM activities pedagogically in the learning environment becomes difficult because teachers do not have sufficient knowledge and experience about STEM components, especially engineering, technology and design (Chai et al., 2019; Faikhamta et al., 2020). According to the results of the research conducted by Ültay and Ültay (2020), the STEM performance of teachers who lack knowledge, skills and pedagogical experiences about STEM activities decreases. Accordingly, it is natural for teachers with low STEM knowledge, skills and pedagogical experience to have low STEM performance in the learning process. However, the point that needs to be pointed out is the evaluation of teachers’ in-service training to support their STEM knowledge and skills. As a result of the related research (Yıldırım and Türk, 2018;Karademir-Coşkun et al., 2020; Papadakis et al., 2021a; Nikolopoulou, 2022a), it is seen that teachers feel inadequate in their knowledge, skills and pedagogical experiences in STEM education.

Furthermore, they could not improve themselves in STEM education since the training they receive in pre-service training is insufficient, and they do not receive in-service training (El-Deghaidy and Mansour, 2015; Karademir-Coşkun et al., 2020). Research results show that teachers’ pedagogical content knowledge (PCK) for STEM education should be supported (Karademir-Coşkun et al., 2020; Papadakis et al., 2021a; Nikolopoulou, 2022a). Based on these findings, teachers must determine and develop their STEM-related pedagogical competencies through in-service training.

There is a certain number of studies examining the STEMPCK skills of teachers and prospective teachers in standard literature (Wang and Fan, 2018; Chai et al., 2019, 2020; Yıldırım and Şahin-Topalcengiz, 2019; Rahman et al., 2022). However, no studies compare the STEMPCK skills of preschool teachers working in different countries, limited to the researchers’ literature review. Nevertheless, it is expected that teachers working in different countries will significantly contribute to the literature by examining their STEMPCK skills because of in-service training for STEM. Making an international comparison of the qualifications of the education received by the teachers in their own countries as a result of STEM education will first reveal the quality of the education received. In addition, comparisons between countries can contribute to preparing STEM teacher training programs. Examining subjects with scientific foundations such as STEM in teacher education in different cultures can reveal countries’ scientific understanding and pedagogical approaches. Ultimately, it provides significant findings for future research for the countries being compared. The standard or different features that will be determined from the STEMPCK comparison of the countries’ teachers can contribute to the teacher education paradigm for STEM education. What should the content of STEM education be given to teachers? What pedagogical practices should teachers do for STEM education? For the answers to these questions to be universal, it is necessary to conduct many studies in different countries. However, it is essential to examine the STEMPCK status of teachers in the countries compared and how teachers receive STEM education are affected by STEMPCK. Indeed, it is necessary to determine the existing situation to predict the validity of international exams and to lay the foundation for the basics of the in-service training programs to be prepared for teachers. In this context, The Program for International Student Assessment (PISA) 2018 exam results and the Organization for Economic Co-operation and Development (OECD) countries’ average scores can be a criterion for comparing STEMPCK teachers of countries with STEM education. PISA Exam Results for Turkey and Greece has given in Table 1 to see the reading, mathematics, and average science results of both countries:

**Table 1:** 2018 PISA exam results for Turkey and Greece*.

Country	Reading average	Mathematics average	Science average
Turkey	466	454	468
Greece	457	451	452
OECD countries	487	489	489

Organisation for Economic Cooperation and Development (OECD, 2019).

It can be seen that Greece scored 457 in reading comprehension, 451 in mathematics, and 452 in science, while Turkey scored 466 in reading comprehension, 454 in mathematics, and 468 in science. The results show that Greece and Turkey remained below the average of OECD countries in reading, mathematics, and science (OECD, 2019). The fact that both Greece and Turkey practice STEM education in early childhood and fall below the OECD countries’ average may be an essential measure of STEM education.

The 2018 PISA test results of Singapore, Hong Kong, China, Finland, Britain, the United States, Germany, and Austria are higher than the average score of OECD countries, and Greece and Turkey are below the average score of OECD countries (OECD, 2019). While there are significant indicators that science education in early childhood education affects the overall results of PISA scores (especially science and mathematics), it can be stated that STEM education also contributes to the success of PISA in these countries. (Havu-Nuutinen et al., 2022). Although STEM education is practised in Greece and Turkey, the fact that it is below the OECD average reveals that countries should be evaluated in terms of having a teacher training policy supported by STEM training (Papadakis et al., 2018, 2019, 2021a; Dorouka et al., 2020; Gözüm and Kandır, 2021; Yıldırım, 2021; Gözüm, 2022; Mercan and Kandır, 2022). What matters here is the STEM pedagogical content knowledge (STEMPCK) of preschool teachers, for it is they who foster attitudes and interest in children who receive STEM education in their early years (Yıldırım and Şahin-Topalcengiz, 2019). This being the case, it is expected that a comparison of the STEMPCK of Greek and Turkish preschool teachers will contribute to the literature and the countries’ teacher training policies concerning STEM education. Therefore, this study aims to compare the STEMPCK scores of Greek and Turkish preschool teachers and examine whether there is a significant difference in the STEMPCK scores of teachers based on the STEM training variable.

## Literature review

### STEM education in early childhood

The preschool years are the critical period for starting education if STEM literacy continues throughout life (Jipson et al., 2014). Early childhood is critical for children’s brain and neuron development. Children’s experiences in the first 8 years of life shape their lives in the years to come. Therefore, it is now acknowledged that starting the learning experience with STEM education in early childhood will yield positive outcomes in the future (Moomaw and Davis, 2010; Torres-Crospe et al., 2014; Allen, 2016). Teachers motivate children in early childhood to compare STEM education activities with real-life problems and produce solutions. Looking for solutions to their problems helps develop children’s sense of curiosity while supporting their research and inquiry skills (John et al., 2018; Tank et al., 2018). Children test themselves on how to apply their experiences in newly encountered situations by learning new information through research. Developing various projects with STEM applications in early childhood supports children’s cognitive, affective and behavioural skills. Children’s skills backed by STEM education cover all the 21st-century skills. It is an adequate area for developing problem-solving, cooperation, creativity, communication, and critical thinking (Israsena et al., 2016; Ludwig et al., 2016; Perignat and Katz-Buonincontro, 2019).

Supporting children’s STEM skills in early childhood classes can be increased by teaching in play settings prepared with a child-centred approach. Early childhood STEM education also lets children artistically develop their skills, self-expression, technology literacy, and engineering skills by playing games in science, mathematics, engineering, and technology (Van Hoorn et al., 2011). Science and the nature of science are mirrored in STEM education. Since early childhood, STEM education has supported children’s interest in and attitude towards all areas of science.

When children’s attitudes towards and interest in science are fostered in their early years, this is reflected positively in their academic achievements in later years. Therefore, children should be given STEM education early (Kershaw et al., 2009). Lamb et al. (2015) integrated STEM education into the syllabuses of kindergarten, second-and fifth-grade children and examined how this affected their cognitive and affective development. The STEM-integrated syllabus was applied from kindergarten to fifth grade. Their study reported that the children’s self-efficacy, interest, and knowledge increased significantly. Teachers play a critical role in integrating STEM education into the syllabuses applied to children. It is essential to determine how teachers integrate STEM education into the syllabus (Chalmers et al., 2017). When teachers are being given STEM training, they need to be given information about teaching practices and the materials required for teacher STEM proficiency and to practice integrated STEM education (Stohlmann et al., 2012). Countries are expected to support teachers through their education policies so they can successfully integrate STEM education into the syllabuses applied to children. Accordingly, if qualified teachers conduct STEM activities with children starting in their early years, their knowledge, skills, and academic achievement will increase, and countries will obtain the trained workforce they require (Quigley and Herro, 2016). From this point of view, the current research results, in which Turkish and Greek teachers’ views on early childhood STEM education are collected, will be examined, and the situations of the countries where the research is conducted will be described.

In the research conducted by Nikolopoulou (2022a), the opinions of Greek teachers about STEM activities for children between the ages of 4–7 were examined. Greek teachers stated that implementing STEM activities supported children’s knowledge, skills and interests in learning. Teachers said they considered children’s needs and motivations, cognitive development levels, and learning outcomes while preparing STEM activities. They also mentioned that the main difficulties with STEM activities were teacher training, lack of infrastructure, limited time to implement the activities, and ensuring children’s interest and active participation. Papadakis et al. (2021a) profiled the attitudes of Greek and pre-service preschool teachers towards using educational robotics in STEM education. They found no significant difference between preschool and pre-service teachers’ attitudes towards using educational robotics. Based on the findings of that study, they discussed the quality of the training to be given to teachers in integrating educational robotics into classrooms. They further recommended revising pre-service teachers’ syllabi to consider new STEM and educational robotics technologies. Yıldırım (2021) asked Turkish preschool teachers their opinions on preparing STEM activities and reported that when preschool teachers carry out STEM activities, they use different methods and techniques depending on the STEM content. Teachers had problems planning lessons for STEM education due to their lack of content knowledge. Teachers’ STEM education practices were also found to support their professional competencies. The study recommends revising professional development and pre-service teacher training programs to support teachers’ STEM competencies and content knowledge.

Research in both countries shows that teachers know the importance of STEM education in early childhood. However, they do not consider themselves sufficient in STEM subjects and applications (Ültay and Ültay, 2020; Yıldırım, 2021; Papadakis et al., 2021a; Nikolopoulou, 2022a). According to the research about STEM education (Ültay and Ültay, 2020; Yıldırım, 2021; Papadakis et al., 2021b; Nikolopoulou, 2022a), PISA 2018 results, Turkey and Greece have the same group of problems with STEM education. Given these results, the STEMPCK of Greek and Turkish preschool teachers who support children’s STEM skills in early childhood should be compared. The first research question in this study is: “*Does STEMPCK of preschool teachers differ between the two countries?*” This question clearly can solve many problems about STEM-oriented teacher education. For example, the results of the preparation, implementation and evaluation of STEM-oriented teacher education programs developed in one country may contribute to the other. In addition, solution suggestions determined in one country to support STEMPCK of teachers can be implemented in another country and contribute to the use of time and finance. This research question will help countries develop STEMPCK policies for teachers who support children’s STEM skills in early childhood. Next, preschool teachers’ STEMPCK is explained in light of the literature.

### STEMPCK theoretical framework

Although there is no consensus on pedagogical content knowledge (PCK) in the literature, this model developed by Shulman (1986) is generally accepted. According to Shulman (1986), PCK is pedagogical, contextual, and content knowledge developed by teachers to support children’s learning. Teachers create their PCKs using their content knowledge and teaching methods when teaching specific content to children. According to Grossman (1990), PCK consists of content, pedagogical, and contextual knowledge. Teachers use content, pedagogical, and contextual knowledge together in the teaching process to form the PCK for teaching a specific topic. In the Technology Pedagogical Content Knowledge (TPACK) model proposed by Mishra and Koehler (2006), PCK is a combination of pedagogical knowledge (PK) and content knowledge (CK). They argue that PCK contains extensive information on how to organize teaching methods and content-specific characteristics appropriate to a particular context for teaching. PCK creates a link between the pedagogy practised by the teacher, the program, and assessment in the children’s learning process. Teachers create ideas for different thinking foundations by using alternative teaching methods to teach specific content. By using PCK effectively, teachers increase children’s prior knowledge and awareness when they have to develop different solutions to the same problems (Mishra and Koehler, 2006).

When PCK for STEM education is examined, the definition of STEMPCK made by Saxton et al. (2014) consists of three elements. The first element of STEMPCK is teachers’ knowledge of considering what they know about STEM content. The second element is teachers’ knowledge of guiding children in the STEM teaching process. The third element is the knowledge of integrating technology into the learning environment to improve teachers’ teaching of STEM content. However, for STEM content to be used effectively in the learning environment, teachers are expected to develop a deep pedagogical understanding of STEM education and the content of STEM education. It is not easy to integrate the disciplines of science, technology, engineering, and mathematics in STEM content and to prepare content for children in line with STEM philosophy (Beswick and Fraser, 2019; Margot and Kettler, 2019). In addition to such difficulties as integrating the disciplines due to the nature of STEM content, the changes occurring today in information and technologies make it difficult for teachers to adapt PCK to the learning process. In this case, teachers’ competencies in using information, communication, and technologies (ICT) are expected to improve (Beswick and Fraser, 2019; Penprase, 2020; Silva et al., 2020; Gözüm, 2022). Teachers are expected to have advanced knowledge and skills concerning 21st-century skills and understanding, which are closely related to the nature of STEM education and are among the learning outcomes. Research results show that teachers with improved knowledge and skills regarding 21st-century skills use it more effectively in the PCK learning process for STEM education (Dede, 2010; Howland et al., 2012; Voogt and Roblin, 2012; Ertuğrul Akyol, 2020). For teachers to provide adequate STEM education to children, the STEMPCK theoretical framework consists of interacting with the nature of STEM content and different combinations of PCK elements. Yıldırım and Şahin-Topalcengiz (2019) completed a literature review of the STEM theoretical framework and argued that it consisted of STEM content information (Moore and Smith, 2014; Srikoom et al., 2017), STEM integration information (Bryan et al., 2016; Türk et al., 2018; Beswick and Fraser, 2019; Margot and Kettler, 2019), pedagogical information (Shulman, 1986; Grossman, 1990; Mishra and Koehler, 2006; Yusof et al., 2012; Saxton et al., 2014), 21st-century skills information (Dede, 2010; Howland et al., 2012; Voogt and Roblin, 2012; Ertuğrul Akyol, 2020) and context knowledge (Shulman, 1986; Barnett and Hodson, 2001). Based on this, the elements of the STEMPCK theoretical framework will be discussed under the title of Preschool Teachers’ STEMPCK.

### Preschool teachers’ STEMPCK

While examining the preschool teachers’ STEMPCK, this paper will explain STEM content knowledge, STEM integration knowledge, pedagogical knowledge, 21stCentury skill knowledge, and context knowledge within the theoretical framework of STEMPCK.

Teachers must know about STEM content if they are to provide early childhood children with practical and qualified STEM education. Teachers need to have a deep knowledge of STEM content areas and the ability to combine knowledge with experience (Whitebook and Ryan, 2011). Çorlu et al. (2015) emphasized that teachers who do not know about STEM content and education will not be able to acquire children’s STEM learning outcomes. Although there is no adoption content in STEM content (Holmlund et al., 2018), it is argued that the integrated delivery of STEM disciplines will be more beneficial for the development of children (Bybee, 2013). However, preschool teachers are expected to be competent in STEM content so they can integrate STEM disciplines and present them to children when teaching them. The study by Moore and Smith (2014) found that teachers used science and mathematics more than technology and engineering when providing STEM education. This research result means that teachers prefer disciplines with STEM content where they feel competent. This result can be considered an essential deficiency in the integrated delivery of STEM education. Knowledge of integration is as necessary as the content of STEM education. The preschool teacher is expected to integrate the subject content pedagogically so that he or she can implement STEM activities in the learning environment (Ostler, 2012). This requires that STEM and PCK combine. A preschool teacher with a good knowledge of STEM components is expected to use his pedagogical knowledge to integrate STEM into early childhood classes. Pedagogical knowledge, which the teacher is expected to have, covers planning activities, implementing them, and evaluating them afterwards. Teachers should have advanced classroom management skills and teaching methods and techniques and be well versed in children’s learning psychology (Shulman, 1986; Briscoe and Peters, 1997).

The National Research Council [NRC] (2014) has described the characteristics of STEM education. According to National Research Council [NCR] (1994), the 21st-century skills of children receiving STEM education are expected to improve. The STEM and PCK knowledge of educators is expected to increase. Equipped with STEM content knowledge and Pedagogical Field Knowledge, the teacher understands the needs of children and aims to furnish them with the skills to possess 21st-century skills by considering the children’s development. The 21st-century skills that teachers possess when implementing STEM activities matter. Teachers are expected to be role models for the 21st-century skills they aim to teach children. These 21st-century skills are considered life and career skills. They are expressed as global awareness, information and media literacy, leadership, responsibility, communication, efficiency, technology literacy, creativity, problem-solving, and critical thinking behaviours (Kennedy and Odell, 2014). Teachers should integrate content by creating context according to the characteristics of their region, the children’s backgrounds, and the region where the schools are found when planning STEM activities (Barnett and Hodson, 2001; Harris and Hofer, 2011; Gkontelos et al., 2022). Sharapan (2012) suggestions for preschool teachers’ context knowledge are as follows: While teachers create a context for STEM, they should select the events, phenomena, and objects children meet in their immediate environment. STEM activities should be built on the events that take place in daily life. Teachers should consider children’s interests and needs when creating context. Meaningful contexts for STEM are crucial as they support children’s learning. Paint, toys, Lego, parks, etc. can create meaningful contexts for children in STEM education. According to Allen (2016), contexts for STEM activities in early childhood should include concrete experiences. Johnson (2016) says that the contexts created in STEM activities should support children’s basic scientific process skills such as asking questions, guessing, observing, and experimenting. Preschool teachers’ knowledge of creating context is expected to affect STEM practices positively. STEMPCK combines teachers’ STEM knowledge, pedagogical knowledge, context knowledge, and 21st-century skills.

When teachers know about content in STEM practices, this makes them more confident in planning and implementing activities (Bers et al., 2013; Eng Tek et al., 2016). Teachers enable multidimensional learning by bringing together different disciplines in STEM content knowledge and forming connections between these disciplines (Smith and Karr-Kidwell, 2000). When preschool teachers experience uncertainty about teaching STEM content, this can cause them to feel anxiety when implementing activities, leading to a reduction in teacher confidence concerning conducting STEM activities and a corresponding drop in the quality and effectiveness of the STEM education activity (Hedlin and Gunnarsson, 2014; Cohrssen and Page, 2016). For teachers to implement STEM activities effectively and proficiently, unique teaching methods and pedagogical skills should be supported by in-service and pre-service training (Atiles et al., 2013; Bers et al., 2013). To support the STEMPCK competencies of teachers who plan and implement STEM activities, teachers should have hands-on practice, and the training they receive should not be theoretical only (Bers et al., 2013; Alexander et al., 2014). When teachers’ STEM knowledge and skills are supported, their beliefs, attitudes, and feelings towards STEM practices can improve and make STEM activities more effective for children (Hedlin and Gunnarsson, 2014; Aldemir and Kermani, 2017; Park et al., 2017).

The study conducted by Koyunlu-Ünlü and Dere (2019) found that when preschool teachers received STEM training, this positively affected their STEM awareness. The study by Chanunan (2021) found that STEM PCK-based training positively affects the STEMPCK of pre-service science and mathematics teachers. The results of this research shed light on the training of teachers equipped with the knowledge and skills to teach STEM. Faikhamta et al. (2020) developed a pedagogical content knowledge-based STEM professional development program and applied it to science teachers. The research found that the implemented program positively supported teachers’ STEM knowledge and practices and developed awareness about STEM disciplines. This being the case, the STEMPCK of preschool teachers in Greece and Turkey who received and did not receive STEM training should be examined. Teachers who received STEM training are expected to have high STEMPCK. This is thought to be necessary for countries’ teacher training policies. Therefore, this study’s second research question is*, “How do preschool teachers’ training influence their STEMPCK*?*”* It is expected that teachers will be supported by STEM training, that children will be provided with sufficient support and infrastructure for STEM education, and that Greece and Turkey will develop economically and technologically. How this situation interacts with the STEM education variable by country is expected to be similar for all the world countries (*Australia*, *Indian* and *Malaysian,* etc.) (Thomas and Watters, 2015). Although the countries where teachers supported by STEM training live vary, support for their STEMPCK competencies should not cause a significant difference. In this case, the study’s research question is *“What is the relation between the teachers’ country of origin and their training in STEMPCK scores?”*

## Materials and methods

### Research model

The primary purpose of this study is to compare the STEMPCK of Greek and Turkish preschool teachers. The research was conducted using the descriptive survey method, a quantitative research approach. The study aimed to describe the STEMPCK status of Greek and Turkish preschool teachers by using the descriptive survey method. The descriptive survey model is used to learn individuals’ attitudes, opinions, beliefs, and demographic characteristics in educational sciences (Johnson and Christensen, 2014).

### Participants

The study participants were 104 Greek and 565 willing Turkish preschool teachers selected using simple random sampling in line with the quantitative survey method. With simple random sampling, each participant has an equal chance of participating in the study. As the participants participated in the study independently of each other, the probability of them representing the universe is high (Büyüköztürk et al., 2011). Table 2 shows the participants’ demographic details by country.

Table 2 shows that a total of 104 preschool teachers from Greece participated in the study, of whom 80.8% (*n* = 84) were female and 19.2% (*n* = 20) were male; 43.3% of the teachers held a bachelor’s degree (*n* = 84), 56.7% held a master’s degree or doctorate. While 34.6% (*n* = 36) of the Greek preschool teachers who participated in the study had received STEM training, 65.4% (*n* = 68) had not. The average age of the Greek teachers participating in the study was 35.09 years. Furthermore, 19.2% (*n* = 20) of the Greek preschool teachers participating in the study taught children aged 36–48 months, 11.5% (*n* = 12) taught children aged 49–60 months, and 69.2% (*n* = 72) taught children aged 61–72 months.

**Table 2:** Participants’ demographic details.

Country		Gender		Education level		Have you received any form of STEM training?		Age						
		Female	Male	Bachelor’s degree	Master or doctorate	Yes	No	20–25	26–30	31–35	36–40	41–45	46 − +	Total
Greek	*n*	84	20	45	59	36	68	7	13	26	48	10	–	104
	%	80.8	19.2	43.3	56.7	34.6	65.4	6.7	12.5	25.0	46.2	9.6	–	100.0
Turkey	*n*	496	69	398	167	210	355	96	119	112	172	45	21	565
	%	87.8	12.2	70.4	29.6	37.2	62.8	17.0	21.1	19.8	30.4	8.0	3.7	100.0
Total	*n*	580	89	443	226	246	423	103	132	138	220	55	21	669
	%	86.7	13.3	66.2	33.8	36.8	63.2	15.4%	19.7	20.6	32.9	8.2	3.1	100.0

A total of 565 preschool teachers from Turkey participated in the study, of whom 87.8% (*N* = 496) were female, 12.2% (*N* = 69) were male, and 70.4% of the teachers held a bachelor’s degree (*N* = 84), and 29.6% held master’s degree or doctorate. While 37.2% (*n* = 210) of the Turkish preschool teachers participating in the study had received STEM training, 62.8% (*n* = 355) had not. The Turkish teachers’ average age in the study was 33.29 years. Of the Turkish preschool teachers participating in the study 10.4% taught children aged 36–48 months (*n* = 59), 28.1% taught children aged 49–60 months (*n* = 159), and 61.4% taught children aged 61–72 months (*n* = 347).

### Data collection tool

The data collection tool used in this study was the STEM Pedagogical Content Knowledge Scale (STEMPCK Scale) developed by Yıldırım and Şahin-Topalcengiz (2019). When they developed their scale, they performed exploratory factor analysis on the data of 443 pre-service teachers and confirmatory factor analysis on the data of 212 pre-service teachers. The construct validity studies for the STEMPCK Scale found six factors, namely, 21st-Century Skills, Pedagogical Knowledge, Mathematics, Science, Engineering, and Technology. The fit index values resulting from the confirmatory factor analysis of the scale revealed a good fit: (CFI = 0.93, NFI = 0.93, GFI = 0.99, AGFI = 0.97, IFI = 0.93, SRMR = 0.043, RMSEA = 0.034). The internal consistency coefficient of the total STEMPCK Scale was found to have a Cronbach’s Alpha value of 0.95. The internal consistency coefficients of the sub-factors of the STEMPCK Scale ranged between 0.78 and 0.90. These results showed that the STEMPCK Scale was valid and reliable data collection tool to measure pre-service teachers’ STEM pedagogical content knowledge.

### Validity and reliability study of the data collection tool

The participants in this study were Turkish and Greek preschool teachers, yet the data collection tool had been developed using pre-service preschool teachers. As the data collection tool’s target group had changed, this study’s data set was used to perform the validity and reliability analyses. Construct validity was examined by subjecting the Greek and Turkish preschool teachers’ data sets to separate CFA. The number of participants (*N*_Greece_ = 104; *N*_Turkey_ = 565) varied by country. Fit index values are affected by the number of participants. Therefore the CFA analysis fit index values to be applied to the data sets for the Greek and Turkish teachers were worked out differently as the data set for the Greek participants is *N* < 250, the Comparative Fit Index (CFI) value, which is less affected by the sample, was examined first. The Normed Fit Index (NFI) and Incremental Fit Index (IFI) values were examined together with the CFI value within the scope of the comparative model fit index. The IFI value matters in that it is calculated by considering the sample size and the complexity of the model. The Goodness of Fit Index (GFI) value, which tests the model regardless of the sample size, is also considered. The Adjustment Goodness of Fit Index (AGFI) value should be examined to decide the fit value adjusted according to the degree of freedom of GFI. It is recommended that the Root Mean Square Error of Approximation (RMSEA) value is preferred less in samples where *N* < 250. Like the RMSEA value, the Standardized Root Mean Square Residual (SRMR) value also does not show a good fit value in small samples. The chi-square value (*X*^2^) is susceptible to sampling. At the same time, the *X*^2^ value is significant in samples where *N* < 250 (*p* > 0.05) is insignificant in large samples (*p* < 0.05). Therefore, it is argued that the value of *X*^2^ divided by the degree of freedom (*df*) or (*X*^2^/*df*) will yield better results to evaluate the model’s overall goodness of fit (Gürbüz, 2019). Therefore, (*X*^2^/*df*), the CFI, NFI, GFI, AGFI, and IFI values were considered in the CFA analysis applied to the Greek data set. By contrast, the Turkish data set considered (*X*^2^/*df*), the CFI, NFI, GFI, AGFI, IFI, SRMR, and RMSEA values because the sample size was sufficient. The same fit index values were examined for the entire data set. Table 3 shows the fit index values (fiv) resulting from the CFA analysis by country.

On examination of Table 3, the *X*^2^ value for the fit index value of the Greek participants’ data set is small and significant (*p* > 0.05), and it is thought that this is because the sample was small (*N* < 250). Examination of the data set for the Turkish and total participants shows the X^2^ value to be large and not significant (*p* < 0.05). This lack of significance may be because the Turkish and comprehensive data set was (*N* > 250). The *X*^2^/*sd* values were examined because fit values for data sets are affected by the number of participants. The *X*^2^/*df* value for the Greek data set was 1.08. Being <3, it showed a good fit. The *X*^2^/*df* value for the Turkish data set was 3.94, and 4.67 for the total participants. *X*^2^/*df* values between 3 and 5 show an acceptable fit (Munro, 2005; Şimsek, 2007; Hooper et al., 2008). When the fit index values of the Greek and Turkish participants are examined, the CFI, NFI, GFI, AGFI, and IFI values range between 0.90 and 0.95, thus showing acceptable fit values. On examination of the fit index values of the data set for the Greek and Turkish participants in total, they were found to be acceptable (Bentler, 1980; Bentler and Bonett, 1980; Baumgartner and Homburg, 1996; Marsh et al., 2006). SRMR and RMSEA values range between.05 and.08, showing an acceptable level (Browne and Cudeck, 1993). Therefore, the STEMPCK Scale was valid at an acceptable level.

**Table 3:** Fit index values as a result of CFA analysis applied to the data set.

Country	*X* ^2^	*df*	*X*^2^/*df*	CFI	NFI	GFI	AGFI	IFI	SRMR	RMSEA
Greek	1587.54	1,469	1.080	0.91	0.92	0.91	0.92	0.92	–	–
Turkey	5793.65	1,469	3.944	0.93	0.90	0.92	0.94	0.93	0.057	0.065
Data	6862.42	1,469	4.671	0.94	0.91	0.92	0.93	0.93	0.060	0.068
Acceptable Fiv		3 < *X*^2^/*df* < 5		90 ≤ CFI, NFI, GFI, AGFI, IFI < 0.95					0.05 ≤ SRMR, RMSEA ≤ 0.08	
Good Fiv		*X* ^2^/*df* < 3		0.95 ≤ CFI, NFI, GFI, AGFI, IFI ≤ 0.99					SRMR, RMSEA ≤ 0.05	

Table 4 shows the reliability values of the STEMPCK Scale and its factors based on the data set of the participants in this study.

**Table 4:** Cronbach’s alpha values for STEMPCK scale and its component factors.

STEMPCK scale and factor	Greek	Turkey	Total data
STEMPCK scale	0.85	0.88	0.90
Pedagogical knowledge	0.82	0.84	0.87
Science knowledge	0.79	0.83	0.85
Technology knowledge	0.78	0.82	0.86
Engineering knowledge	0.81	0.85	0.87
Mathematical knowledge	0.77	0.79	0.84
21st-century skills knowledge	0.80	0.83	0.91

Table 4 shows that when the Cronbach’s Alpha (α) values of the data set of Greek and Turkish participants are examined, the Cronbach’s Alpha (*α*) value of STEMPCK Scale and Its Factors is more significant than 0.70. The Cronbach’s Alpha (*α*) values for the STEMPCK Scale and its Factors for the Greek and Turkish participants’ total data set a range between.84 and.91. According to George and Mallery (2003), a Cronbach’s Alpha value of 0.7 ≤ α <0.9 shows that the scale is reliable in terms of internal consistency. Therefore, the STEMPCK Scale and Its Factors used in the study can be considered reliable.

### Data collection

The researchers converted the data collection tools into Google Forms and had them published in Greek and Turkish. The researchers then distributed the Google Forms to the participants using social media apps such as Facebook and WhatsApp. The institutions where the participants worked did not assist in distributing the Google Forms. The Google Form includes a consent form for the participants to complete saying they are voluntarily participating in the study. It also includes an ethics declaration, saying that the participant’s data will not be shared. The researchers shared their email addresses and contact information on Google Forms so the participants could obtain information about the research and ask questions. After the participants approved the consent form saying they wanted to participate voluntarily in the study, they shared personal information and fill in the data collection tool’s fields. All the scale items on the Google Form had the mandatory box selected to avoid data loss and were completed accordingly. The Google Form also included an open-ended question for those teachers who did not want to have to fill in the scale items and those who wanted to express their opinions about the comprehensibility of the scale items. The participants voiced no negative opinions about the mandatory items or their comprehensibility. This may indicate that the participants filled in the Google Form without social desirability bias.

All procedures performed in studies involving human participants were following the ethical standards of by Kafkas University Ethics Committee in Turkey - Türkiye research committee (document no: E.30529/24–05.2022) and by the University of Crete ethics committees in Greece (document no: 606/18-5-2022) and also the 1964 Helsinki Declaration and its later amendments or comparable ethical standards.

### Data analysis

This study’s data were analyzed for two different reasons. The purpose of the analyses was to determine the validity and reliability of the data collection tool. The purpose of the second analysis was to determine the findings for the research problems. Validity and reliability analyses were performed by dividing the data for the Greek and Turkish participants into two sets. CFA and internal consistency coefficients were calculated for both data sets. Validity and reliability analyses were made for the data set, which was the sum of the data of the Greek and Turkish participants. AMOS and SPSS computer software programs were used for these statistical operations. The mean, standard deviation, percentage, and frequencies were calculated as required by descriptive analysis. A MANOVA test was performed for the research problem to examine whether the dependent variables had a significant difference over the independent variable. Tukey, a *post hoc* test, was used to determine the direction in cases where significant differences were found. An independent t-test was conducted to see if there was a significant difference based on whether the Turkish and Greek participants received STEM training in their respective countries.

### Data analysis assumptions

The assumptions of the CFA and MONOVA tests were examined when the data were analyzed. The Greek, Turkish, and complete data sets were analyzed separately when examining the DFA analysis assumptions. The data for the Greek and Turkish participants were analyzed as a single data set when conducting the MANOVA test.

Before conducting Confirmatory Factor Analysis, multiple normality values were examined in the Greek and Turkish participants’ data set. The CR values for the Greek and Turkish participants in the data set were below 10, and the kurtosis values for the data sets ranged between −3 and + 3. The multiple normality values for the participants’ data set show normal distribution. It can therefore be argued that the multiple normality assumption is met in the CFA analysis (Gürbüz and Şahin, 2018).

When conducting the MANOVA test, Box’s M test values were examined to test whether the variance and covariance matrices were equal. It was understood that the variance and covariance matrices of the dependent variables of *country* and *teachers receiving STEM training* were equal (Box’s *M* = 88.304, *F* = 1.075, *sd*_1_ = 28, *sd*_2_ = 18447.287, *p* = 0.374). Levene’s test results were examined to determine whether the variance distributions of independent variables were homogeneous. The Levene test results showed that the scale and the factors were homogeneous as there was no significant difference from *p* > 0.05. Wilks’ Lambda values were examined for the multivariate test when performing MANOVA analyses.

### Findings

This section gives the findings related to the research questions. Table 5 gives the findings to the research question, “*Does STEMPCK of preschool teachers differ between the two countries?”*

**Table 5:** Descriptive statistic and multivariate test.

Scale / Component	Country	Q*	*N*	Mean	SD	
Pedagogical knowledge	Total	Yes	246	53,45	6,41	
		No	423	51,62	5,81	
		Total	669	52,29	6,10	
Science knowledge	Total	Yes	246	33,14	4,60	
		No	423	29,28	5,80	
		Total	669	30,70	5,70	
Technology knowledge	Total	Yes	246	28,31	5,16	
		No	423	25,32	4,96	
		Total	669	26,42	5,23	
Engineering knowledge	Total	Yes	246	27,30	5,27	
		No	423	24,28	4,59	
		Total	669	25,39	5,06	
Mathematical knowledge	Total	Yes	246	34,07	5,12	
		No	423	30,90	5,86	
		Total	669	32,06	5,80	
21st century skills knowledge	Total	Yes	246	18,67	1,88	
		No	423	17,77	2,25	
		Total	669	18,10	2,16	
STEMPCK	Total	Yes	246	194,95	20,74	
		No	423	179,20	20,09	
		Total	669	184,99	21,69	
*Multivariate test*						
Effect	*λ*	*F*	Hypothesis *df*	Error *df*	Sig.	*η*_p_^2^
Intercept	0.018	6102.288	6.000	660.000	0.000	0.982
Country	0.985	1.678	6.000	660.000	0.124	0.115
STEM training	0.919	9.724	6.000	660.000	0.000	0.181
Country* STEM training	0.991	.974	6.000	660.000	0.442*	0.009

* Have you received any form of STEM training?

Since multiple Anovas have been made in Tables 6 a type I error is possible. Therefore, Bonferroni correction is used to check type 1 error significant difference. Since Bonferroni correction was used in this study, each ANOVA was evaluated at a significance level (p) of.017.

**Table 6:** Multidirectional analysis of variance values and pairwise comparisons.

Tests of between-subjects effects							
Corrected Model	Dependent variable	Sum of squares	*df*	Mean Square	*F*	Sig.	*η*_p_^2^
	Pedagogical knowledge	617.862	3	205.954	5.650	0.001	0.025
	Science knowledge	2410.044	3	803.348	27.619	0.000	0.111
	Technology knowledge	1445.485	3	481.828	18.977	0.000	0.079
	Engineering knowledge	1418.467	3	472.822	19.972	0.000	0.083
	Mathematical knowledge	1569.295	3	523.098	16.583	0.000	0.070
	21st-century skills knowledge	134.909	3	44.970	9.986	0.000	0.043
	STEMPCK	39223.572	3	13074.524	31.602	0.000	0.125
Intercept	Pedagogical knowledge	871610.382	1	871610.382	23909.836	0.000	0.973
	Science knowledge	304730.901	1	304730.901	10476.483	0.000	0.940
	Technology knowledge	225074.817	1	225074.817	8864.833	0.000	0.930
	Engineering knowledge	211683.467	1	211683.467	8941.520	0.000	0.931
	Mathematical knowledge	335863.933	1	335863.933	10647.077	0.000	0.941
	21st-century skills knowledge	107254.320	1	107254.320	23817.931	0.000	0.973
	STEMPCK	11070067.491	1	11070067.491	26757.427	0.000	0.976
Country	Pedagogical knowledge	64.365	1	64.365	1.766	0.184	0.003
	Science knowledge	76.319	1	76.319	2.624	0.106	0.004
	Technology knowledge	53.517	1	53.517	2.108	0.147	0.003
	Engineering knowledge	2.081	1	2.081	0.088	0.767	0.000
	Mathematical knowledge	3.168	1	3.168	0.100	0.751	0.000
	21st-century skills knowledge	5.630	1	5.630	1.250	0.264	0.002
	STEMPCK	621.205	1	621.205	1.502	221	0.002
STEM Training	Pedagogical knowledge	117.720	1	117.720	3.229	0.073	0.005
	Science knowledge	1275.617	1	1275.617	43.855	0.000	0.062
	Technology knowledge	715.516	1	715.516	28.181	0.000	0.041
	Engineering knowledge	652.426	1	652.426	27.559	0.000	0.040
	Mathematical knowledge	718.045	1	718.045	22.762	0.000	0.033
	21st-century skills knowledge	95.974	1	95.974	21.313	0.000	0.031
	STEMPCK	18346.808	1	18346.808	44.346	0.000	0.063
Country * STEM Training	Pedagogical knowledge	60.798	1	60.798	1.668	0.197	0.003
	Science knowledge	4.043	1	4.043	0.139	0.709	0.000
	Technology knowledge	0.066	1	0.066	0.003	0.959	0.000
	Engineering knowledge	3.993	1	3.993	0.169	0.681	0.000
	Mathematical knowledge	4.777	1	4.777	0.151	0.697	0.000
	21st-century skills knowledge	6.184	1	6.184	1.373	0.242	0.002
	STEMPCK	52.228	1	52.228	0.126	0.722	0.000
Error	Pedagogical knowledge	24241.944	665	36.454			
	Science knowledge	19342.946	665	29.087			
	Technology knowledge	16884.103	665	25.390			
	Engineering knowledge	15743.354	665	23.674			
	Mathematical knowledge	20977.542	665	31.545			
	21st-century skills knowledge	2994.556	665	4.503			
	STEMPCK	275123.427	665	413.719			
Total	Pedagogical knowledge	1854591.000	669	Corrected total	Pedagogical knowledge	24859.806	668
	Science knowledge	652506.000	669		Science knowledge	21752.990	668
	Technology knowledge	485515.000	669		Technology knowledge	18329.587	668
	Engineering knowledge	448591.000	669		Engineering knowledge	17161.821	668
	Mathematical knowledge	710550.000	669		Mathematical knowledge	22546.837	668
	21st-century skills knowledge	222449.000	669		21st-century skills knowledge	3129.465	668
	STEMPCK	23210502.000	669		STEMPCK	314346.99	668
*Pairwise comparisons*							
	Dependent variable	(I)	(J)	Mean difference (I-J)		Std. error	Sig.
	Pedagogical knowledge	Yes	No	1.214		0.675	0.073
	Science knowledge	Yes	No	3.996^*^		0.603	0.000
	Technology knowledge	Yes	No	2.993^*^		0.564	0.000
	Engineering knowledge	Yes	No	2.858^*^		0.544	0.000
	Mathematical knowledge	Yes	No	2.998^*^		0.628	0.000
	21st-century skills knowledge	Yes	No	1.096^*^		0.237	0.000
	STEMPCK	Yes	No	15.153 ^*^		2.276	0.000

(I)/(J) = Have you received any form of STEM training?

“*Does STEMPCK of preschool teachers differ between the two countries?*” When the findings of this research question are examined, Table 5 shows (λ = 0.985, *F*_(6.660)_ = 1.678, *p* = 0.124, *p* > 0.01) showing no significant difference in STEMPCK scores between countries.

*“How do preschool teachers’ training influence their STEMPCK?”* When the findings of the research question are examined, the multivariate test results in Table 5 show (*λ* = 0.919, F _(6.660)_ = 9.724, *p* = 0.000, *p* < 0.01), indicating a significant difference in teachers’ STEMPCK scores based on whether they received STEM training or not. When the total STEMPCK score and STEMPCK factors are examined, a significant difference is found in the Pedagogical knowledge factor (*F*_(1.665)_ = 3.229, *p* = 0.073, *p* > 0.01), science knowledge (*F*_(1.665)_ = 43.855, *p* = 0.000, *p* < 0.01), technology knowledge (*F*_(1.665)_ = 28.181, *p* = 0.000, *p* < 0.01), engineering knowledge (*F*_(1.665)_ = 27.559, *p* = 0.000, *p* < 0.01), mathematical knowledge (*F*_(1.665)_ = 22.762, *p* = 0.000, *p* < 0.01), 21st-Century Skills knowledge (*F*_(1.665)_ = 21.313, *p* = 0.000, *p* < 0.01) and STEMPCK total score (*F*_(1.665)_ = 44.346, *p* = 0.000, *p* < 0.01).

*“What is the relation between the teachers’ country of origin and their training in terms of STEMPCK scores?”* When the findings of the research question are examined, the multivariate test results in Table 5 show (*λ* = 0.991, *F*_(6.660)_ = 0.974, *p* = 0.442, *p* > 0.01), indicating no significant difference in the teachers’ STEMPCK scores.

A significant difference is found between the teachers’ STEMPCK scores based on whether they received STEM training or not (Table 6). The direction of significant difference is examined in Table 6.

When the direction of significant difference in Table 6 is examined, a significant difference is found in the science knowledge (*p* < 0.01), technology knowledge (*p* < 0.01), engineering knowledge (*p* < 0.01), mathematical knowledge (*p* < 0.01), 21st-century skills knowledge (*p* < 0.01) factors and the STEMPCK total score (*p* < 0.01) in favor of teachers who received STEM training.

The figures below were used to examine by country if there was a significant difference between the STEMPCK scores based on whether or not the teachers had received STEM training by looking at the estimated marginal means values. Also examined was whether the Turkish and Greek teachers receiving STEM training or not made a significant difference in their respective countries. The STEMPCK and sub-factors are examined in Table 7.

**Table 7:** Independent *t*-test result of STEM education status by country.

	Country	Q*	*N*	Mean	*sd*	*df*	*t*	*p*
Pedagogical knowledge	Greece	Yes	36	52,94	5,23	102	3.308	0.000
		No	68	50,60	3,82			
		Total	104	51,41	4,33			
	Turkey	Yes	210	53,71	6,57	563	3.807	0.000
		No	355	51,62	6,12			
		Total	565	52,40	6,36			
Science knowledge	Greece	Yes	36	32,50	3,10	102	5.134	0.000
		No	68	28,27	4,38			
		Total	104	29,74	4,45			
	Turkey	Yes	210	33,25	4,81	563	7.720	0.000
		No	355	29,48	6,03			
		Total	565	30,88	5,89			
Technology knowledge	Greece	Yes	36	27,63	5,09	102	3.279	0.001
		No	68	24,61	4,10			
		Total	104	25,66	4,67			
	Turkey	Yes	210	28,42	5,17	563	6.630	0.000
		No	355	25,46	5,11			
		Total	565	26,56	5,32			
Engineering knowledge	Greece	Yes	36	26,97	4,87	102	3.306	0.001
		No	68	24,33	3,21			
		Total	104	25,25	4,04			
	Turkey	Yes	210	27,35	5,35	563	7.042	0.000
		No	355	24,27	4,82			
		Total	565	25,42	5,23			
Mathematical knowledge	Greece	Yes	36	33,69	4,95	102	2.768	0.007
		No	68	30,94	4,75			
		Total	104	31,89	4,97			
	Turkey	Yes	210	34,13	5,16	563	6.479	0.000
		No	355	30,89	6,06			
		Total	565	32,10	5,95			
21st Century Skills knowledge	Greece	Yes	36	19,13	1,53	102	2.963	0.004
		No	68	17,76	2,54			
		Total	104	18,24	2,33			
	Turkey	Yes	210	18,59	1,92	563	4.477	0.000
		No	355	17,77	2,19			
		Total	565	18,08	2,13			
STEMPCK	Greece	Yes	36	191,88	12,18	102	5.439	0.004
		No	68	177,54	13,10			
		Total	104	182,50	14,46			
	Turkey	Yes	210	195,48	21,85	563	8.558	0.000
		No	355	179,52	21,16			
		Total	565	185,45	22,75			

*Have you received any form of STEM training?

Table 7 shows a significant difference in the pedagogical knowledge scores of Greek preschool teachers based on whether they received STEM training or not (*t*_102_ = 3.308; *p* = 0.000; *p* < 0.050). There is a significant difference in Turkish preschool teachers’ pedagogical knowledge scores based on whether they received STEM training or not (*t*_563_ = 3.807; *p* = 0.000, *p* < 0.050). When the direction of the significant difference was examined, it was found to favour the pedagogical knowledge of preschool teachers who received STEM training.

There is a significant difference in Greek preschool teachers’ science knowledge scores based on whether they received STEM training or not (*t*_102_ = 5.134; *p* = 0.000, *p* < 0.050). There is a significant difference in Turkish preschool teachers’ science knowledge scores based on whether they received STEM training or not (*t*_563_ = 3.807; *p* = 0.000, *p* < 0.050). When the direction of the significant difference was examined, it was found to favour the science knowledge of preschool teachers who received STEM training.

There is a significant difference in the technology knowledge scores of Greek preschool teachers based on whether they received STEM training or not (*t*_102_ = 3.279; *p* = 0.001; *p* < 0.050). There is a significant difference in Turkish preschool teachers’ technology knowledge scores based on whether they received STEM training or not (*t*_563_ = 3.807; *p* = 0.000, *p* < 0.050). When the direction of the significant difference is examined, it is found to favour the technical knowledge of preschool teachers who received STEM training.

There is a significant difference in the engineering knowledge scores of Greek preschool teachers based on whether they received STEM training or not (*t*_102_ = 3.279; *p* = 0.001; *p* < 0.050). There is a significant difference in Turkish preschool teachers’ engineering knowledge scores based on whether they received STEM training or not (*t*_563_ = 3.807; *p* = 0.000, *p* < 0.050). When the direction of the significant difference is examined, it is found to favour the engineering knowledge of preschool teachers who received STEM training.

There is a significant difference in the mathematical knowledge scores of Greek preschool teachers based on whether they received STEM training or not (*t*_102_ = 3.279; *p* = 0.007; *p* < 0.050). There is a significant difference in Turkish preschool teachers’ mathematical knowledge scores based on whether they received STEM training or not (*t*_563_ = 3.807; *p* = 0.000, *p* < 0.050). When the direction of the significant difference is examined, it is found to favour the mathematical knowledge of preschool teachers who received STEM training.

There is a significant difference in 21st-century skills knowledge scores of Greek preschool teachers based on whether they received STEM training or not (*t*_102_ = 2.963; *p* = 0.004; *p* < 0.050). There is a significant difference in 21st-century skills knowledge scores of Turkish preschool teachers based on whether they received STEM training or not (*t*_563_ = 4.477; *p* = 0.000, *p* < 0.050). When the direction of the significant difference was examined, it was found to favour the 21st-century skills knowledge of preschool teachers who received STEM training.

There is a significant difference in the STEM PCK scores of Greek preschool teachers based on whether they received STEM training or not (*t*_102_ = 5.439; *p* = 0.004; *p* < 0.050). There is a significant difference in Turkish preschool teachers’ STEMPCK scores based on whether they received STEM training or not (*t*_563_ = 8.558; *p* = 0.000, *p* < 0.050). When the direction of the significant difference was examined, it was found to favour the STEMPCK scores of Greek and Turkish preschool teachers who had received STEM training.

## Discussion and conclusions

The primary purpose of this study is to compare the STEMPCK of Greek and Turkish preschool teachers. The study data were examined to determine whether there was not a significant difference between Greek and Turkish preschool teachers in STEMPCK and its components (Pedagogical knowledge, science knowledge, technology knowledge, engineering knowledge, mathematical knowledge, and 21st-century skills knowledge; Table 5). The results show that Greek and Turkish teachers have similar levels of pedagogical content knowledge concerning STEM education. According to the 2018 PISA exam results, Turkey and Greece scored below the OECD average for reading, science, and mathematics, and their respective average scores were similar. The interpretation of the results reveals that there is no significant difference between Greek and Turkish preschool teachers’ STEMPCK scores and that this is consistent with the 2018 PISA results (OECD, 2019). However, the fact that Turkey and Greece’s 2018 PISA results are below OECD countries indicates that children’s STEM knowledge and skills are not adequately supported. However, getting quality STEM education early can enable children to influence their academic success in the following years. At this point, it is expected that their teachers will have developed STEMPCK so that children can receive quality STEM education. According to the results of the research (Ültay and Ültay, 2020; Yıldırım, 2021; Papadakis et al., 2021a; Nikolopoulou, 2022a) conducted with Greek and Turkish preschool teachers, it has been determined that there are PCK deficiencies in STEM education.

According to the results of the study by Ling et al. (2020), in which they detected teachers’ PCK shortcomings concerning STEM education, the teachers were found to have PCK shortcomings regarding STEM. Given the results of this research, teachers should receive STEM training to address their PCK shortcomings. Among those influential variables in identifying STEM needs and addressing them, one key variable is teachers receiving STEM training. To this end, the study examined *“How do preschool teachers’ training influence their STEMPCK?”* The participating teachers receiving STEM training significantly differed in the total STEMPCK score (Table 6). Both Greek and Turkish preschool teachers receiving STEM training resulted in a significant differentiation in STEMPCK scores. Another result of the research is that there is no significant difference in the interaction between the country variable and the STEM training variable. When the STEM training rates of the teachers participating in the study were examined, it was seen that 34.6% of the Greek participants (*n* = 36) and 37.2% of the Turkish participants (*n* = 210) received STEM training. Given these results, a critical topic of discussion is how practical STEM activities will be in the classrooms of those Greek and Turkish teachers who have not received STEM training. Teachers who do not receive STEM education will likely experience difficulties planning, implementing and evaluating STEM activities. As a result of supporting Greek and Turkish teachers with STEMPCK, they can make an adequate STEM education. It is recommended that teachers take STEM education, according to the literature.

In their qualitative research, Weng et al. (2020) held semi-structured interviews with teachers. They concluded that teachers lacked STEM knowledge and PCK for STEM education. They recommended that teachers attend STEM training courses to increase their STEMPCK. The study conducted by Shernoff et al. (2017) identified professional needs concerning STEM based on the opinions of teachers and administrators. The teachers said that they considered themselves inadequate in terms of STEMPCK. They stated that they did not receive enough STEM training before service or in-service and did not know enough about planning, implementing, and assessing activities. Preschool teachers in Greece and Turkey should be given support in the form of STEM training before service and in-service. Though there are science, mathematics and technology courses related to STEM education, there are no specific courses in the integrated STEM content when the content of the preschool teacher training program in Turkey is examined. The same issue is also observed in Greece. Although preschool teachers in Greece have classes on STEM, they do not take classes for integrated STEM education.

Nonetheless, the most basic STEMPCK of teachers should be given in the pre-service period. Current developments and new approaches to STEM should be supported by training during the service process, and STEMPCK developments should be provided. Furthermore, the STEMPCK deficiencies of teachers are so much higher than in the pre-service period that it may be necessary to make intense efforts to eliminate them during the service period. Hence, it is not a coincidence that these countries are below OECD, according to PISA results. However, it should be stated that having a STEM education does not make the STEM teaching process efficient because STEM education is a complex education process. PCK support of teachers alone is also not enough for STEM education. At the same time, it has been determined that teachers need training for STEM content knowledge. Teachers’ lack of STEM content and PCK knowledge may reduce their tendency to avoid STEM activities or to engage children in STEM activities. When the relevant literature is examined,

The study conducted by Hsu et al., 2011 examined primary school teachers’ perceptions of engineering, design, and technology concerning STEM. They determined that while teachers had strong beliefs in engineering, design, and technology, they did not feel competent enough in knowledge or educational practices. Training is vital if teachers are to feel competent in STEM content knowledge. Teachers can practice STEM education effectively by receiving training and increasing their self-efficacy beliefs (Rahman et al., 2017; Ling et al., 2020). The STEM content knowledge of Greek and Turkish teachers who receive and do not receive STEM training differs significantly from those who receive STEM training (see Table 6). According to Bandura (1986), teachers need the first-hand experience to increase their self-efficacy. Therefore preschool teachers’ STEM training background is critically important. The study by Yılmaz (2019) determined that pre-service teachers with STEM experience were interested in STEM education and tended to engage in more STEM activities than those without STEM experience. The STEM education of teachers from Turkey and Greece should be beyond just the cognitive acquisition of STEM content and pedagogy. STEM education supports children’s learning outcomes by influencing teachers’ self-efficacy in organizing and conducting STEM activities. Teachers’ doing STEM activities has an effect that improves their self-efficacy in this stage (Nathan et al., 2011). Teachers’ STEM self-efficacy affects not only their current STEM education experiences but also their past direct experiences with STEM readiness. Other studies point out preschool teachers receive less training in the STEM education discipline than primary and secondary education teachers (Aldemir and Kermani, 2017). This literature information is valid for both Greek and Turkish preschool teachers. Therefore, preschool teachers need to acquire the necessary content information in the pre-service period to do STEM activities in their educational processes. Mathematics and science course hours of pre-service teachers in pre-service programs play an influential role in their STEM education. The lack of teachers’ science and mathematics courses may be the main reason teachers have difficulties providing quality STEM education (King et al., 2013) because teachers should have the content knowledge to make STEM education effective. In this study, the significant differentiation in the STEM content knowledge of the teachers who received STEM education is in line with the literature results. Wan et al., (2021) argue that preschool teachers must be given STEM training during teacher training because preschool teachers have little experience in carrying out STEM activities involving multidisciplinary issues. Considering that Greek and Turkish preschool teachers have similar STEMPCK, STEM training should be given to preschool teachers of both countries as a matter of urgency, and their experiences should be supported. Papadakis et al. (2021a) and Yıldırım (2021) recommend that in-service training programs for STEM be provided for both Greek and Turkish preschool teachers and pre-service teachers and that STEM be included in teacher training programs. Another significant result of this study is that STEMPCK and its components differ significantly in favour of Greek and Turkish teachers who received STEM training (Table 7).

Papadakis et al. (2021a) and Yıldırım (2021) state that although it is helpful to provide teachers with STEM training, this is not enough to integrate STEM into classroom practices. The development of STEM content and pedagogy content knowledge of Turkish and Greek preschool teachers makes classroom STEM activities more understandable and suitable for child development (Williams, 2016). Therefore, education programs that include STEM content and pedagogical knowledge should be prepared for preschool teachers who are intended to provide STEM education (Schuster et al., 2012). It is understood that teachers who receive STEM education apply more qualified STEM activities to children. However, the teacher does not explain all aspects of STEM education which influence children’s academic success and learning in STEM education (Fore et al., 2015). Training that will make teachers experienced in STEM activities is considered critical in the tendency to do STEM education because the STEMPCKs of teachers who receive STEM education differ significantly. This situation has essential effects on the reflection of STEM activities in practice. Thus, the results of this research are in line with the literature.

Wang et al. (2011) cited the lack of a STEM education program specifically tailored for teachers and children as the reason for not implementing qualified STEM activities in the classroom. Considering that there is no vocational training program specially prepared to train Greek and Turkish pre-service preschool teacher, it can be thought that Greek and Turkish teachers face similar difficulties in STEM activities. It is believed that implementing programs that support preschool teachers’ knowledge, skills, and experiences in STEM, not just in Greece and Turkey but also those teaching early childhood classes in other countries too, will significantly affect how those countries develop (Cunningham and Higgins, 2015). Greece and Turkey can be considered representative examples here.

Moore et al. (2014) argue that the STEM training programs to be prepared for Greek and Turkish preschool teachers should be prepared in line with the contextual content for the conditions of the country where the teachers are located and the locations of the schools where they work (urban, rural and suburban). In-service training programs to be developed for different learning environments can meet specific pedagogical needs. It should not be forgotten that preschool teachers should plan and implement STEM activities according to the needs of the children. This shows that teachers’ pedagogical knowledge concerning STEM practices according to the cultures of the different countries where children live is just as crucial as preschool teachers’ STEM knowledge and experience. For example, according to Sullivan and Bers (2018), children aged 3–6 years do not all possess the same programming skills for use in STEM activities. The cultural effects of countries on children’s programming skills are observable. The average programming skill scores of children aged 3–6 years studying in Singapore kindergartens may match the average programming skill scores of first- and second-grade children in America. Cultural differences should therefore be considered when developing STEM and vocational education programs for Greek and Turkish preschool teachers and their children. The following should be considered when designing educational programs: teachers and pre-service teachers should gain practical experience in authentic learning environments where STEM education is done for children and develop their pedagogical knowledge to plan STEM content activities based on children’s development levels and learning needs (Brenneman et al., 2019).

Teacher training and in-service programs to be prepared in support of Greek and Turkish preschool teachers’ STEMPCK should be current, consistent, and developed in line with studies and practices in specific developmentally appropriate cultures (Papadakis et al., 2018, 2019, 2021a,b; Dorouka et al., 2020; Gkontelos et al., 2021; Gözüm and Kandır, 2021; Yıldırım, 2021; Gözüm, 2022; Mercan and Kandır, 2022). In addition, Greek and Turkish preschool teachers should be provided with resources prepared by experts in STEM education, and blogs, social media, and video sharing platforms should be created for more accurate and effective practices (Early Childhood STEM Working Group, 2017; Gözüm, 2021).

STEM education relates to 21st-century skills because teachers provide up-to-date information and support some skills. In this research, teachers’ knowledge of 21st-century skills was also examined within the scope of STEMCPK. The present study found that Greek and Turkish preschool teachers receive STEM training, positively affecting their 21st-century skills. It is expected that the 21st-century skills of Turkish and Greek teachers are developed to provide qualified STEM education. STEM education programs that will include 21st-century skills should be developed starting from the pre-service period to support the 21st-century skills of Turkish and Greek teachers and to develop their practice attitudes towards STEM education. For teachers to be effective in STEM activities, they are expected to be technology literate, which is crucial in the digital age. The study by Çetin and Kahyaoğlu (2018) investigated how the application of STEM activities affected pre-service teachers’ attitudes towards STEM education and 21st-century skills. The study concluded that pre-service teachers’ attitudes towards STEM education and 21st-century skills improved in classes where STEM activities were applied. In the preschool years, when teachers are role models for children, the development of 21st-century skills of teachers can enable more effective implementation of STEM practices in the classroom. Therefore, the fact that Turkish and Greek preschool teachers are equipped with 21st-century skills enables them to acquire cognitive and affective skills that will support PCK. The mixed research study by Ertuğrul Akyol (2020) examined the effect of STEM activities on pre-service teachers’ 21st-century skills. It concluded that STEM activities positively affected pre-service teachers’ problem-solving, computational, critical, and creative thinking skills. The qualitative results showed that pre-service teachers with 21 t century skills effectively used simple materials in robotic and coding-based STEM activities. Teachers’ role is essential in using digital applications in STEM activities in classroom applications (Papadakis et al., 2021b; Nikolopoulou, 2022b).

Teachers with advanced 21st-century skills are expected to be able to adapt technology to STEM activities. Moreover, Turkish and Greek preschool teachers can cope with many factors that prevent them from including educational robotics (Papadakis et al., 2021b) or mobile applications (Nikolopoulou, 2021) in classroom STEM activities due to their advanced 21st-century skills. Support for teachers’ 21st- century skills will help them to plan and implement quality STEM activities and assess them. One of the goals of STEM education is for children to produce creative solutions to daily life problems because of technology-based learning and STEM activities supported by technology (Dede, 2010; Howland et al., 2012). Furthermore, increased teacher knowledge of 21st-century skills will support the development of children’s skills such as problem-solving, collaborative learning, creative thinking, and self-learning through STEM activities (Dede, 2010; Voogt and Roblin, 2012; Gkontelos et al., 2022). In this context, teachers’ knowledge of 21st-century skills from a STEM standpoint must be supported if they are continuously open to innovation and integrate technology and other variables into learning environments. This is why 21st-century skills should be included in the training programs that support STEMPCK.

Consequently, it was determined that Turkish and Greek teachers were similar to STEMPCK, and STEMPCK positively supported teachers who received STEM education in both countries. Another critical result of the research is that the country variable does not affect STEM education. In this context, a joint emergency action plan can be prepared to support STEMPCK of Turkish and Greek preschool teachers. The researchers believe that the Results and Discussion part of this study will help shape the content and structure of the vocational education planned for preschool teachers.

### Recommendations

Preschool teachers in Greece and Turkey have similar countries and plan teacher training programs and in-service training activities together, considering cultural factors. Furthermore, they can prepare projects jointly with preschool STEMPCK. Greek and Turkish preschool teachers can take advantage of being neighbourly teachers from a country with an above-average PISA score to investigate the STEMPCK profiles of Greek and Turkish preschool teachers and prepare urgent training programs. Teachers must examine countries’ early childhood education policies and invest in technology-supported applications to provide adequate education using STEMPCK.

### Limitations and future research

One fundamental limitation of the study is the number of teachers participating in the study from Greece and Turkey. The results of this study are limited to the participating teachers. Although the number of participants is thought to be good when the ratio of preschool teachers working in their countries is considered, future studies can be planned with more participants. Nevertheless, despite these limitations, it is expected that comparing the STEMPCK of Greek and Turkish teachers will contribute to future research. Future research could benefit from mixed research models combining qualitative and quantitative research methods to add more detail to the results of this study.

## Data availability statement

The original contributions presented in the study are included in the article/supplementary material, further inquiries can be directed to the corresponding author.

## Ethics statement

The studies involving human participants were reviewed and approved by all procedures performed in studies involving human participants were following the ethical standards of by Kafkas University Ethics Committee in Turkey - Türkiye research committee (document no: E.30529/24-05.2022) and by the University of Crete ethics committees in Greece (document no: 606/18-5-2022) and also the 1964 Helsinki Declaration and its later amendments or comparable ethical standards.. Written informed consent to participate in this study was provided by the participants' legal guardian/next of kin.

## Author contributions

AG: writing, literature review, methodology, design, data collecting, data analysis, data validation, conclusion and discussion. SP: literature review, methodology, data collecting and editing. MK: literature review, design, and data collecting. All authors contributed to the article and approved the submitted version.

## Conflict of interest

The authors declare that the research was conducted in the absence of any commercial or financial relationships that could be construed as a potential conflict of interest.

## Publisher’s note

All claims expressed in this article are solely those of the authors and do not necessarily represent those of their affiliated organizations, or those of the publisher, the editors and the reviewers. Any product that may be evaluated in this article, or claim that may be made by its manufacturer, is not guaranteed or endorsed by the publisher.
